# Transitional Care in Patients With Hirschsprung Disease: Those Left Behind

**DOI:** 10.1097/DCR.0000000000003208

**Published:** 2024-04-23

**Authors:** David S. Thompson, Joseph R. Davidson, Kathryn E. Ford, Stavros P. Loukogeorgakis, Simon Eaton, Simon C. Blackburn, Joe Curry

**Affiliations:** 1 Department of Neonatal and Paediatric Surgery, Great Ormond Street Hospital for Children, London, United Kingdom; 2 Stem Cells and Regenerative Medicine Section, UCL-GOS Institute of Child Health, London, United Kingdom

**Keywords:** Hirschsprung disease, Pediatric surgery, Transition, Transitional care

## Abstract

**BACKGROUND:**

The long-term effects of Hirschsprung disease are clinically variable. An improved understanding of challenges patients may face as adults can help inform transitional care management.

**OBJECTIVE:**

To explore the outcomes and transitional care experiences in adult patients with Hirschsprung.

**DESIGN:**

Cohort study.

**SETTING:**

Single center.

**PATIENTS:**

All patients treated for Hirschsprung between 1977 and 2001 (aged older than 18 years at the time of survey distribution in July 2018–2019). Eligible patients were sent validated multidomain surveys and qualitative questions regarding their transitional care.

**MAIN OUTCOME MEASURES:**

Status of transitional care, bowel function, and quality-of-life assessment. Qualitative analysis of transitional care experience.

**RESULTS:**

Of 139 patients, 20 had received transition care (10 had at least 1 visit but had been discharged and 10 were receiving ongoing follow-up). These patients had inferior bowel function and quality-of-life scores at follow-up. Twenty-three patients (17%) had issues with soiling at the time of discharge, and 7 patients received transitional care. Of these 23 patients, 9 (39%) had a normal Bowel Function Score (17 or more), 5 (22%) had a poor score (less than 12), and 1 had since had a stoma formation. Eighteen patients (13%) had active moderate–severe issues related to bowel function, only 5 had been transitioned, and just 2 remained under ongoing care. Importantly, when these patients were discharged from our pediatric center, at a median age of 14 (interquartile range, 12–16) years, 10 of 17 patients had no perceptible bowel issues, suggesting a worsening of function after discharge.

**LIMITATIONS:**

The retrospective design and reliance on clinical notes to gather information on discharge status as well as patient recall of events.

**CONCLUSIONS:**

There remains a small but significant proportion of Hirschsprung patients for whom bowel function either remains or becomes a major burden. These results support a need to better stratify patients requiring transitional care and ensure a clear route to care if their status changes after discharge. See **Video Abstract**.

**ATENCIÓN DE TRANSICIÓN EN PACIENTES CON ENFERMEDAD DE HIRSCHSPRUNG, LOS QUE SE QUEDAN ATRÁS:**

**ANTECEDENTES:**

Los efectos a largo plazo de la enfermedad de Hirschsprung son clínicamente variables. Una mejor comprensión de los desafíos que los pacientes pueden enfrentar cuando sean adultos puede ayudar a informar la gestión de la atención de transición.

**OBJETIVO:**

Explorar los resultados y las experiencias de atención de transición en pacientes adultos con Hirschsprung.

**DISEÑO:**

Estudio de cohorte.

**AJUSTE:**

Unico centro.

**PACIENTES:**

Todos los pacientes tratados por Hirschsprung 1977-2001 (edad >18 años en el momento de la encuesta, Julio de 2018-2019). A los pacientes elegibles se les enviaron encuestas multidominio validadas, así como preguntas cualitativas sobre su atención de transición.

**PRINCIPALES MEDIDAS DE RESULTADOS:**

Estado de la atención de transición, función intestinal y evaluación de la calidad de vida. Análisis cualitativo de la experiencia de cuidados transicionales.

**RESULTADOS:**

De 139 pacientes, 20 habían recibido atención de transición (10 tuvieron al menos una visita pero habían sido dados de alta y 10 estaban recibiendo seguimiento continuo). Estos pacientes tenían puntuaciones inferiores de función intestinal y calidad de vida en el seguimiento. Veintitrés (17%) pacientes tuvieron problemas para ensuciarse en el momento del alta y 7 recibieron atención de transición. De estos, 9/23 (39%) tenían una puntuación de función intestinal normal (≥17), 5/23 (22%) tenían una puntuación baja (<12) y un paciente había tenido desde entonces una formación de estoma. Dieciocho (13%) pacientes tenían problemas activos de moderados a graves relacionados con la función intestinal, solo cinco habían realizado la transición y solo 2 permanecían bajo atención continua. Es importante destacar que cuando estos pacientes fueron dados de alta de nuestro centro pediátrico, a una edad promedio de 14 [RIQ 12-16] años, 10/17 no tenían problemas intestinales perceptibles, lo que sugiere un empeoramiento de la función después del alta.

**LIMITACIONES:**

El diseño retrospectivo y la dependencia de notas clínicas para recopilar información sobre el estado del alta, así como el recuerdo de los eventos por parte del paciente.

**CONCLUSIÓN:**

Sigue existiendo una proporción pequeña pero significativa de pacientes con Hirschsprung para quienes la función intestinal permanece o se convierte en una carga importante. Estos resultados respaldan la necesidad de estratificar mejor a los pacientes que requieren atención de transición y garantizar una ruta clara hacia la atención si su estado cambia después del alta. (*Traducción—Dr. Yesenia Rojas-Khalil*)

In recent years, the outcomes for patients with Hirschsprung disease (HSCR) have been increasingly studied; a significant minority of patients experience soiling and fecal incontinence with an associated reduction in quality of life (QoL).^1–4^ In addition, there remains a risk of Hirschsprung-associated enterocolitis, with reported lifetime incidence ranging from 11% to 44%^5,6^; an emerging link with the development of IBD is now recognized.^7^

Studies during the initial childhood years after corrective pull-through surgery show that most children do not appear to demonstrate improvements in their functional status.^2,8^ Adult patients have demonstrably better function, although 10% to 20% of patients have a poor outcome, with fecal incontinence, soiling, and poor psychosocial function compared to age-matched controls.^1,5,8–11^ More recently, impacts in other functional domains have been noted, including urological and sexual function; fertility outcomes in female patients may also be impacted.^12–14^

Recognition of the negative impact on long-term health and well-being beyond childhood highlights the need for continued care in these patients and for transitioning pediatric patients with chronic health conditions into adult services.^15,16^ Regrettably, clinical practice often falls short despite our understanding of the increased need for transition.^15,17–20^ There is no standardized pathway for transitioning patients with congenital GI surgical pathology in the United Kingdom. Little is known about what happens to the patients who are not transitioned, and understanding the path patients take after discharge from pediatric services helps inform how transitional care should be managed.

This was a cross-sectional study of a single-center cohort with 2 aims. First, we performed an analysis of cross-sectional outcomes, comparing adult patients who received transitional care with those who did not. Second, we performed a thematic analysis of the written experiences of patients who were transitioned to gain insight into this process. We hypothesized that patients who have been offered transition care in the past might have worse functional and QoL outcomes, whereas those without may have improved bowel symptoms and psychosocial functioning.

## MATERIALS AND METHODS

This was a cross-sectional cohort study using mixed methods of all patients treated as children between 1977 and 2001 for HSCR at a single regional center. Historically, the department was one of the busiest in the United Kingdom, serving most of North London with a considerable catchment area extending to the north. There has also been a considerable number of referrals for revision surgery from across the country. Ethics approval was obtained from the National Health Service Research and Ethics Committee (No. 17/LO/1692). Patients were identified through clinical coding (International Classification of Diseases-9 751.3 and International Classification of Diseases-10 Q43.1), and case notes were reviewed to confirm a diagnosis. Patients with histologically confirmed HSCR under the care of the surgery team for any treatment were eligible for inclusion.

All patients, meeting these inclusion criteria and currently living within the United Kingdom were invited to take part in a comprehensive follow-up questionnaire (see Appendix 1 at http://links.lww.com/DCR/C330 and Appendix 2 at http://links.lww.com/DCR/C331), which was either emailed or mailed to them at home, and responses were collected from July 2018 to 2019. All included patients were adults at the time of survey distribution. The questionnaire included validated, multidomain surveys including the Bowel Function Score (BFS), Gastrointestinal Quality of Life Index (GIQLI), and Short Form 36 (SF-36).^12,21^ Results from this cohort have been published previously.^3^ The current study reports novel data relating to patients’ experience of care after discharge from pediatric surgery and how the transition process may relate to their functional outcomes.

Recruitment was based on the Strengthening the Reporting of Observational Studies in Epidemiology statement. The lead investigator (J.R.D.) was not involved in the clinical care of any patients. Written consent was obtained from all patients.

### Exclusion Criteria

Eligible adult patients were excluded from this particular analysis if they had died, moved abroad, or were unable to be contacted (no contact information or inappropriate to contact). Exclusions were also applied to those who were unable to complete the questionnaire for language reasons or had a learning disability related to a recognized syndrome or neurodevelopmental disability, which would preclude consent or completing the survey without an advocate.

### Study Setting

Before 2001, there was no formal standardized transition practice at our center and patients (including the present study's patients who were treated between 1977 and 2001) would be referred to adult services at the pediatric surgeon’s discretion. There were a total of 9 consultant pediatric surgeons caring for HSCR patients in the department during this period. For the purposes of this study, transitional care has been retrospectively defined as at least 1 appointment within the gastroenterology or colorectal surgery team immediately after discharge from pediatric surgery, which was arranged and coordinated at the discretion of the lead surgeon responsible for the patient’s care at our institution.

### Definitions

Based on individual recall and narrative, patients were defined as either having no transition service (NTS) or being referred to transition service (TS). TS patients were further divided into 3 categories: 1) single transition clinic appointment and discharged, 2) more than 1 visit but since discharged, or 3) ongoing care in adult services.

Details regarding the patients’ functional status at discharge from pediatric services were retrieved retrospectively from clinical case notes at our institution, usually the final clinic letter. Binary outcomes were defined thus: social continence, need for rectal enemas, and requirement for a stoma or antegrade colonic enema (ACE). Functional status at the time of the study was assessed on the basis of the questionnaire responses.

### Statistical Analysis (Quantitative)

Statistical analyses were performed using Prism version 9.0 (GraphPad) and SPSS version 26.0 (IBM) to delineate function at discharge compared to present day and to compare scores of both BFS and GIQLI between TS and NTS groups as well as the component scores of the SF-36—calculated by Z-score transformation from normal population data.^22,23^ Data are presented as median (interquartile range [IQR]) or mean (SD). Distributions were compared with appropriate parametric and nonparametric tests, with Bonferroni correction for 2 comparisons when comparing component scores of the SF-36. A *p* value of <0.05 was considered statistically significant.

### Qualitative Analysis

In addition to specific bowel function and QoL data, patients were asked open-ended questions relating to their transitional care and long-term follow-up (full questionnaires are included in Appendix 1 at http://links.lww.com/DCR/C330 [male patients] and in Appendix 2 at http://links.lww.com/DCR/C331 [female patients]). Qualitative analysis was performed on the basis of a grounded theory of data collection and evaluation.^24^ Answers with insufficient content for analysis were excluded. Any responses to blank space questions that were answered with sufficient depth were reviewed using an inductive approach, independently reviewed by 2 authors with some experience in qualitative data handling (D.S.T. and J.R.D.; both male, pediatric surgical trainees), and categorized as positive, negative, or neutral. Discrepancies were blindly reviewed by a third independent reviewer with more extensive experience in qualitative research (K.F.; female, pediatric surgeon). Findings were reviewed by supervisors with qualitative research experience (J.C. and S.B.: male, pediatric surgeons; and S.E.: male, scientist). Further thematic analysis was performed to define common aspects of positive and negative experiences. Ultimately, themes were used to generate a proposed model of positive transition.

## RESULTS

After exclusions were applied, 340 patients were approached to take part in the survey, of whom 216 responded (64%); 139 of these patients were adults without a learning disability and therefore eligible for analysis (median age 29 [IQR, 25–34] years; see Fig. S1 at http://links.lww.com/DCR/C332). Of these, 98 (71%) were men and the majority (104; 75%) had rectosigmoid disease (Table 1). Twenty patients were transitioned from the pediatric surgery clinic into adult services. Of those who were transitioned, only 10 were still receiving ongoing follow-up at the time of the survey. Patients reported seeing both gastroenterology (ie, medical GI) and general/colorectal surgery specialists, as well as 1 female patient who saw a pelvic floor specialist after childbirth.

**TABLE 1.** Demographics and outcomes

Variable	*Overall (N = 139*)	*TS (N = 20*)	*NTS (N = 119*)
Sex, male, n (%)	98 (71)	11 (55)	87 (73)
Rectosigmoid, n (%)	104 (75)	12 (60)	92 (77)
Redo operation, n (%)	27 (19)	6 (30)	21 (18)
Last clinic information			
Age at last clinic, y, mean (IQR)	14 (11–16)	16 (10–17)	14 (11–16)
Stoma	3	1	2
ACE	3	0	3
Soiling	24	7	17
Rectal enemas	3	1	2
Redo pull through	27	6	21
Information at survey			
Age at survey, y, mean (IQR)	29 (25–34)	27 (22–33)	29 (26–34)
Current stoma	4	2	2
Current ACE	1	0	1
Weekly/daily soiling	14	3	11
Weekly/daily accident	5	1	4
Severe psychological issues	8	3	5

Data presented as n unless otherwise indicated. ACE = antegrade colonic enema (ie, by appendicostomy or caecostomy); IQR = interquartile range; NTS = no transition services; TS = transition services.

### Status at Discharge

Median age at discharge was similar for the TS and NTS groups (median 16 [IQR, 10–17] vs 14 [IQR, 11–16] years), and the median year of discharge was 2002 for both. At the time of discharge from the pediatric surgery department, 3 patients had a stoma (2%; 1 TS), 3 had an ACE (2%; no TS), and 3 were using rectal enemas (2%; 1 TS). Twenty-four patients (17%) had ongoing issues with soiling and social continence at the time of discharge, of whom 22 were managing their symptoms with oral medications or diet and 2 with rectal enemas. Only 7 of the 24 patients (30%) with soiling and social continence issues had received transitional care (Table 1).

### Status at Survey

**FIGURE 1.**
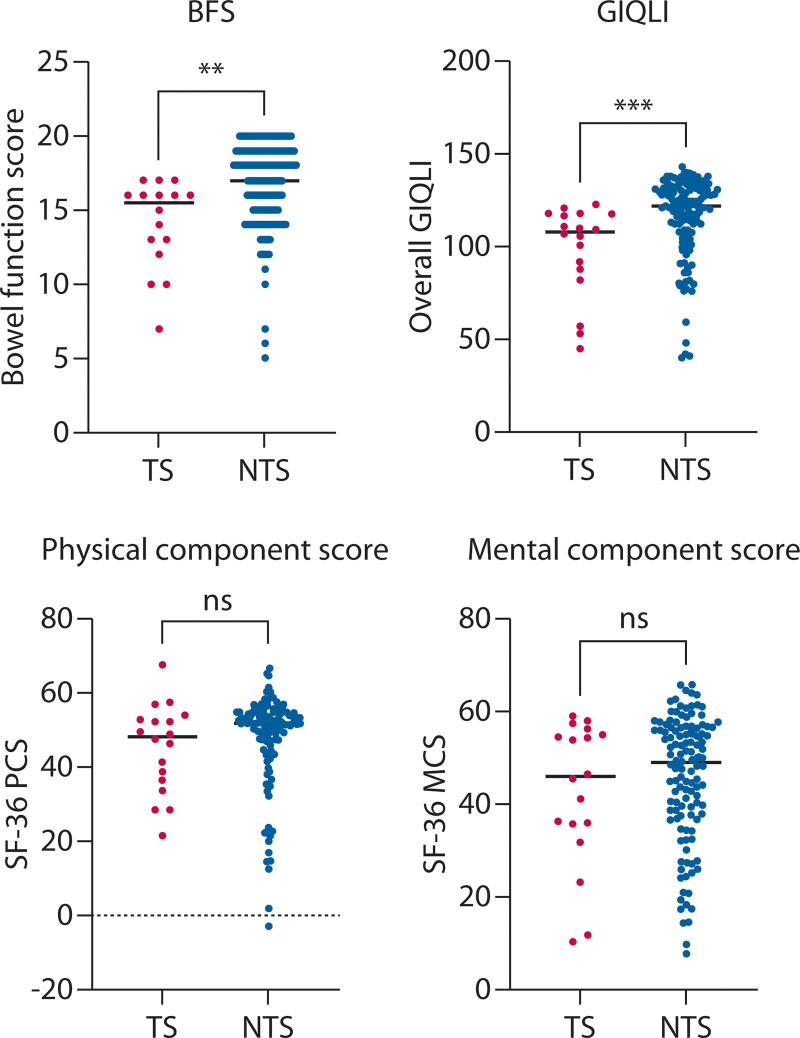
Comparison of functional and quality-of-life outcomes. BFS = Bowel Function Score; GIQLI = Gastrointestinal Quality-of-Life Index; MCS = mental component score; ns = not significant; NTS = no transition services; PCS = physical component score; SF-36 = Short Form 36 (general quality of life questionnaire); TS = transition services. **p < 0.001; ***p < 0.0005.

The median age at the time of the survey was 29 years (NTS vs TS: 29 [25–34] years vs 26 [22–33.75] years, *p* = 0.13). One TS patient had a stoma formed as an adult. Overall, BFS was found to be higher in the NTS group compared to the TS group (17 [15–19] vs 15.5 [12.25–16], *p* = 0.0006; BFS scores were not applied to patients with ACE or stoma). Similarly, the GIQLI scores were higher in the NTS group (122 [108–131] vs 108 [86.5–118], *p* = 0.0004; Fig. 1). There was no difference in either physical (52.0 [47.1–54.8] vs 48.4 [36.0–53.1], *p* = 0.17) or mental (49.18 [37.6–56.5] vs 46.1 [34.9–55.4], *p* = 0.47) component scores (where 50 represents the mean of a matched healthy population) of the SF-36 surveys (Fig 1.).

Of the 3 NTS patients with an ACE at discharge, 2 were later closed and 1 remained. An additional 3 patients had new ACEs formed but subsequently closed under the adult team. There were no ACE patients in the TS group. Three patients underwent major abdominal surgery in adulthood for adhesional obstruction and were all part of the NTS. One TS patient had a new stoma formed.

Of the 24 patients with soiling or social continence issues at discharge, 9 (38%) had BFSs defined as “normal” (BFS 17 or more) and 5 (21%) had poor bowel function (BFS less than 12 or active ACE or stoma). Of the 17 NTS patients with soiling issues at discharge, 5 (30%) reported ongoing soiling issues and 12 (71%) improved. However, 6 NTS patients who were previously well reported new soiling issues emerging in adulthood.

Daily or weekly soiling issues were actively experienced by 14 patients: 3 TS (15%) and 11 NTS (9%). A total of 8 patients reported severe psychological issues related to bowel function: 3 TS (15%) and 5 NTS (4%). Overall, 18 patients (12.9%) had frequent soiling or severe psychological issues related to bowel function. Five of these 18 patients had been transitioned, and only 2 remained under ongoing care. Importantly, when these patients with ongoing issues were last seen in the pediatric clinic, 10 of 18 (55.6%) reported no continence or bowel function issues, suggesting a deterioration after discharge.

### Qualitative Data

Qualitative data were extracted from the blank space questions from all transitioned patients with 100% sufficient for content analysis (20/20). When commenting on the transition process, 7 patients (35%) reflected positively. Seven patients had negative reflections (35%) and 6 responses were deemed neutral (30%).

Quotations from which categories and themes were extracted are shown in Table 2. Data saturation was reached for some but not all of the categories, specifically “challenges of a new environment,” “loss of an important relationship,” and “living with a chronic condition.” Categories could be grouped into themes: “prior planning,” “preparedness,” and “new life beyond pediatric care”—with some categories such as “health literacy” cutting across these.

**TABLE 2.** Extraction of themes and thematic analysis

*Meaning unit*	*Category*	*Theme*
“It was very smooth” “Well-orchestrated and carefully managed” “Smooth and straightforward, there wasn’t long waiting times” “Transition was seamless” “I haven’t had any consistent care”	Structure and organization	Prior planning
“Did not feel rushed” “Slowly introduce myself to the new team” “At no point did I feel like I was being ‘kicked out’ ”	Slow and deliberate process	Prior planning
“It was discussed before what would happen” “It was hard to leave but they assured me that everything will carry on… this made the transition a lot more comfortable”	Active communication before transitioning	Patient preparedness
“I felt very prepared” “I have more control over my health” “I do feel I’m on my own with the disease with no real knowledge of how things have moved on”	Patient health literacy + empowerment	Patient preparedness
“Once transferred, they were fully aware of my medical history and it was just like picking up where I left off” “I had an initial appointment with the consultant just to clarify all the information provided was correct and up to date” “My GP was not really sure what was involved. I always had to recap” “They had a very fixed idea of how things would improve, however this did not help” “I found the experiences [unencouraging] and stressful”	Receiving clinicians prepared	Health services preparedness
“I was referred to a consultant. In all honesty, he had no knowledge of Hirschsprung” “Didn’t feel they understood my problems”	Adult clinicians with expertise	Health services preparedness
“I found it hard to recover in adult wards…. I was only 18 [years old]”	Challenges of a new environment	Adjusting to adulthood
“I felt sad to leave GOSH and my current surgeon as I felt I could trust him and he knows me so well” “Sure you can pass on notes but it’s not the same as long-standing history”	Loss of important relationship	Adjusting to adulthood
“I wanted to try and live with my condition and put it all behind me” “I had lots of investigations and then decided that was about as far as I could go”	Living with a chronic condition	Adjusting to adulthood

Comments from positive responses in green, negative in red, and neutral in dark gray.

GOSH = Great Ormond Street Hospital; GP = general practitioner.

### Prior Planning

The process of transition was a frequent theme. When this was felt to be structured, this was associated with positive experiences: “it was very smooth,” “well-orchestrated and carefully managed,” and “the transition was seamless.” However, negative experiences included a lack of “consistent care.”

A slow speed of the process was also associated with positive experiences, with patients reporting they “did not feel rushed” and “at no point feeling they were being ‘kicked out.'”

### Patient and Health Services Preparedness

Preparedness and understanding appeared most frequently as a theme across all answers and related to both expertise and understanding in the patient as well as in their treating clinical teams.

Patient preparedness for the transition process came in the form of active and consistent communication and assurances from their pediatric team and empowerment from the perspective of patient education and enabling access to patient records (ie, through an online patient portal). Experiences with Health Services were positive when information and understanding was actively sought: “they were fully aware of my medical history and it was just like picking up where I left off,” “I had a meeting with the consultant to clarify all the information was correct and up to date.” There were examples of poor preparedness among Health Services, with a lack of disease-specific knowledge and understanding being a prominent feature of several responses, as well as “fixed ideas” of treatment that might have been performed from other conditions.

### Adjusting to Adulthood

This theme was undersaturated and tended to feature only negative and neutral experiences. One patient commented on the difficulties of recovering in an adult ward: “I found it hard to recover in adult wards ... I was only 18 [years old].” The loss of a trusted clinician and the difficulties of meeting a new team was also a strong theme: “After I received the most incredible care… my transition was never going to be easy.” “I felt I could trust him [pediatric surgery consultant] and he knows me so well.” Two patients described their fatigue with health care management after transition: “I wanted to live with my condition and put it behind me” and “that was about as far as I could go.”

## DISCUSSION

In the present article, we describe patients' transitional care experiences and outcomes during a 35-year period for our institution. At the point of discharge, 17% (n = 24) of our patients had issues with soiling and continence, but at the point of survey, 39% (n = 9) of these patients now had normal BFS. Indicating a continued functional improvement through adulthood. At the point of the survey, 13% (n = 18) had frequent soiling or severe psychological issues related to bowel function. Twenty patients received some form of transitional care; these patients had poorer BFS and GIQLI scores than nontransitioned patients in the survey. Several patients had not received transitional care and had clearly developed new functional issues since their discharge. Qualitative analysis of patients’ transitional pathway experiences revealed prominent themes of planning the process and preparing both the patient and their receiving team. The theme of adjusting to life outside of pediatric services was also noted.

Patients who received transitional care appointments tended to present with lower functional and QoL scores at follow-up. This suggests that the more symptomatic individuals were offered transitional care services, and these more complex patients continued to experience functional issues into adulthood, with poorer long-term outcomes and a higher symptom burden. However, we did identify that some adolescent patients with social continence issues were not offered transitional care but may have benefited from it. Furthermore, the clinical status of patients with HSCR may change after discharge: from the 102 NTS without soiling issues, 6% reported new issues in the survey. Published data from our own cohort and others suggest that adults tend to have better outcomes than children after surgery, indicating a general improvement in function. However, longitudinal assessments are rarely reported.^8,25^

Prominent themes that emerged from qualitative analysis surrounded the planning and preparation of the transitional process, which has been echoed by patients elsewhere with congenital cardiac disease.^26^ Patients also highlighted a lack of experience with Hirschsprung disease among adult services. Adult surgeons or GI specialists with a dedicated interest in these patients would certainly be of great value, although delivering this may be challenging considering the move from centralized pediatric surgery services to more dispersed adult general surgical care. This could drive adult care of congenital GI conditions toward more centralized specialist services, as is seen with congenital cardiology in the United Kingdom (where surgeons and cardiologists continue to see the same patients throughout life). Structured patient and parent education during adolescence and instilling personal ownership of their condition could prevent patients from feeling isolated and empower them to seek help or referral where needed.

Combining objective outcomes and patient-reported experience adds insight into the patient journey. Modern services advocate a more active transition process for lifelong conditions,^15,27^ and this has been well demonstrated in other pediatric disciplines such as the treatment of IBD,^28^ congenital cardiac disease,^29^ and rheumatological conditions.^30^ Historically, consensus differed, and this is reflected in our cohort, where just 14% of patients were transitioned at the lead surgeon’s discretion. The timing of transition has been highly debated and is likely to vary depending on geographical region and local services. In the United Kingdom, it has been suggested that the transition process should begin around the age of 14 years, with no definitive end point.^31^ Many pediatric centers have the ability to continue to see select patients until the age of 25 years, allowing for flexibility within the system.

Transition is an ever-evolving area of health care, and our findings highlight the need to reconsider how we handle these patients. We suggest a formal criterion-based assessment of patients during a transition period that will vary depending on clinical status and suggest a categorized transition accordingly:

1. Those with high symptom burden (ie, ongoing soiling, use of long-term stoma, or antegrade or retrograde enemas) are referred for routine ongoing follow-up to an adult specialist service, ideally one with an interest in and exposure to this patient population, with the aim of long-term follow-up. This may include a period of combined pediatric and adult cover or a specific adolescent clinic with both adult and pediatric surgeon involvement. The final adult team may be a gastroenterologist or colorectal surgeon, but the important factor is clinical expertise and exposure to this specific patient population.
2. Those with moderate symptom burden are referred for a single follow-up with the most appropriate regional adult care team as they approach the upper limit of pediatric age range of 16 to 18 years in the United Kingdom.
3. Those with the least symptom burden are referred to the care of their general practitioner, with sign-posting to relevant adult services provided to the patient and general practitioner and a care summary document provided to both.

We suggest that multidomain, objective screening tools such as BFS and GIQLI should be used to assess both functional status and QoL and to identify those who require referral to adult services. This would formalize a full and systematic review at the initiation of transition and potentially identify individuals with subtle functional concerns. It would also provide a touchstone for referring and accepting clinical teams. Because of the part-retrospective and cross-sectional nature of our review, we are unable to recommend a specific cutoff for the instruments used, but this would be interesting for further work.

It is important to reemphasize that a significant minority of patients in group 3, who are well at discharge, will deteriorate and require referral to adult services later in life. Increasing age has previously been identified as an independent risk factor for poor bowel function in HSCR patients in the only study to report objective outcomes of patients into their fifth and sixth decade of life.^4^ It is essential that all HSCR patients are made aware of the potential for deterioration that may be directly attributable to their condition and that they are empowered to seek referral if needed in the future. Education within the patient community has been the focus of some work by the UK–based Tracheoesophageal Fistula Support Charity (TOFS), a patient-directed charity that publishes patient information aimed at enabling adult patients with tracheoesophageal fistula and VACTRL association to recognize new symptoms as part of their condition with the aim to “encourage self-advocacy, promote wellness, and improve health outcomes.”^32^ Similarly empowered and educated patients with HSCR would be better positioned to recognize and flag changes in their health related to HSCR and seek medical help. This may require input from the pediatric team long after discharge to ensure the appropriate transfer of relevant information to the treating adult specialist. This highlights the ongoing duty of pediatric surgeons to care for their patients, even after discharge.

Limitations of this study include its retrospective nature and reliance on clinic notes to gather information on discharge status, which is open to omission and reporting bias. Furthermore, patient recall was relied on for information about events surrounding their care at discharge or transition, which can be unreliable. Although our survey gave the opportunity for patients to express their opinions and experience of care after discharge from the pediatric service, this lacked a formal qualitative interview. More information would be gleaned from formal interviews and focus groups.^15^

## CONCLUSION

This study provides an opportunity for a longitudinal assessment of patients with HSCR disease and a review of their transitional care. A small but significant number of patients were discharged with active soiling issues, and although some patients will improve after discharge, some will inevitably deteriorate. We identified that bowel function can be a major burden affecting the QoL of patients well beyond their time under pediatric services. This highlights the need for formal and more widespread transitional care.

Although this study focuses on Hirschsprung disease, it suggests that many patients with congenital anomalies require lifelong support. It is therefore important to establish a clear transition protocol, to identify which patients will benefit, and to safety-net others to ensure a clear route to care if their status changes after discharge. This will require strengthening links to adult specialties to provide care for these complex patient cohorts. We believe that transitional care is a process and not a single moment in time. As pediatric surgeons, our duty of care does not end when the patient becomes an adult, and maintaining clear communication between adult and pediatric services is essential in the ongoing care of these patients.

## ACKNOWLEDGMENTS

The authors would like to thank all of the patients who took time to partake in this study and share their experiences. J.R.D., S.P.L., and S.E. acknowledge support from the Great Ormond Street Institute of Child Health NIHR Biomedical Research Centre.

## Supplementary Material
