# Supplementary material for: Transitional Care in Patients With Hirschsprung Disease: Those Left Behind
Source: Dis Colon Rectum. 2024 Apr 23;67(7):977–84. doi: 10.1097/DCR.0000000000003208 (PMC11163890; doi:10.1097/DCR.0000000000003208)
Supplement: Supplementary file 2 [file dcr-67-977-s002.pdf]

Long-Term Outcomes in Hirschsprung's Disease (GOSH)
Questionnaire for adult patients
V1.1 18/09/2017

Open questionnaire

Could you tell us about your transition from care at Great Ormond Street to adult services?

Where do you currently see a specialist for follow up of your Hirschsprung's Disease?

Have you had any further operations since you transitioned into adult care, including a stoma formation or Botox injections?

Do you currently have a stoma (colostomy or ileostomy)?

Yes ☐

No ☐

Do you currently take any medication for constipation, such as Movicol or Lactulose?

Do you take any other medication for any other reason?

Do you have any food allergies or intolerances?

What is your current height and weight?

Height? cm Weight? kg

Do you know of any family members who have Hirschsprung's Disease?

Long-Term Outcomes in Hirschsprung's Disease (GOSH)
Questionnaire for adult patients
V1.1 18/09/2017

<u>Bowel function - Rintala score</u>

**1. You feel when you need to pass stool**	Always	Most of the time	Uncertain	Never	
	☐	☐	☐	☐	
**2. You are able to hold back passing stool**	Always	Problems less than once a week	Weekly problems	No voluntary control	
	☐	☐	☐	☐	
**3. You need to pass stool:**	Less than once every 2 days	Once every 2 days	Once a day	Twice a day	More than twice a day
	☐	☐	☐	☐	☐
**4. You have problems with faecal soiling (staining of the underwear):**	Never	Less than once a week, change of underwear needed only rarely	More than once a week, change of underwear often needed	Daily, requiring protective aids (i.e. pads or diapers)	
	☐	☐	☐	☐	
**5. You have accidents involving stool:**	Never	Less than once a week	Weekly, requiring protective aids	Daily, requiring protective aids day and night	
	☐	☐	☐	☐	
**6. Regarding constipation**	No constipation	Constipation you manage with diet alone	Constipation you manage with medication	Constipation you manage with enemas	
	☐	☐	☐	☐	
**7. Do you suffer from involuntary gas leakage (passing wind without realising)?**	Never	Less than once a week	More than once a week	Daily / constantly	
	☐	☐	☐	☐	
**8. Social impact of bowel function**	No impact	Some impact (i.e bad smells sometimes)	Problems restricting social activities	Major social or psychological problems as a result of bowel function	
	☐	☐	☐	☐	

Please continue on the next page

9. Have you ever had enterocolitis (bowel inflammation) related to Hirschsprung's Disease?

Yes ☐/ No ☐

10. Have you had an episode of enterocolitis in the past year?

Yes ☐/ No ☐

11. Have you ever had recurrent enterocolitis (<u>more than 4 episodes in one year</u>?)

Yes ☐/ No ☐

Is there anything further you would wish to tell us about?

Urinary Function Questionnaire

**1. Have you ever had a urinary tract infection (UTI)?**	No	Yes	If yes, how many UTI in the past year?	
	☐	☐		
**2. How many times do you pass urine each day?**	1 – 3 times	4 – 8 times	More than 8 times	
	☐	☐	☐	
**3. Do you ever need to strain to start/continue urination?**	Never	Rarely	Often	Always
	☐	☐	☐	☐
**4. Does you ever get a sudden urge to pass urine?**	Never	Rarely	Often	Always
	☐	☐	☐	☐
**5. Is the urge so strong that urine escapes before reaching the toilet?**	Never	Rarely	Often	Always
	☐	☐	☐	☐
**6. Does urine ever leak upon straining (e.g.laughing, sneezing or coughing)?**	Never	Rarely	Often	Always
	☐	☐	☐	☐
**7. Does urine ever leak without physical activity or apparent need to urinate?**	Never	Rarely	Often	Always
	☐	☐	☐	☐
**8. Do you ever have night-time wetting (bedwetting)?**	Never	Less than once a week	More often than once a week	Every night
	☐	☐	☐	☐
**9. Do you consider yourself to have social problems due to urinary incontinence?**	No	Yes, due to daytime urinary incontinence only	Yes, due to night-time urinary incontinence only	Yes, due to daytime and night-time urinary incontinence
	☐	☐	☐	☐

Gastrointestinal Quality of Life Index (GIQLI) – please answer in reference only to the time period in the question

**1. How often in the past 2 weeks have you experienced pain in the abdomen**	All of the time	Most of the time	Some of the time	A little of the time	Never
	☐	☐	☐	☐	☐
**2. How often in the past 2 weeks have you experienced a fullness in the upper abdomen**	All of the time	Most of the time	Some of the time	A little of the time	Never
	☐	☐	☐	☐	☐
**3. How often in the past 2 weeks have you experienced bloating / gassy**	All of the time	Most of the time	Some of the time	A little of the time	Never
	☐	☐	☐	☐	☐
**4. How often in the past 2 weeks have you been troubled by excessive wind/flatulence/farting**	All of the time	Most of the time	Some of the time	A little of the time	Never
	☐	☐	☐	☐	☐
**5. How often in the past 2 weeks have you been troubled by strong burping / belching?**	All of the time	Most of the time	Some of the time	A little of the time	Never
	☐	☐	☐	☐	☐
**6. How often in the past 2 weeks have you been troubled by gurgling noises in the abdomen/tummy**	All of the time	Most of the time	Some of the time	A little of the time	Never
	☐	☐	☐	☐	☐
**7. How often in the past 2 weeks have you been troubled by frequent bowel movements?**	All of the time	Most of the time	Some of the time	A little of the time	Never
	☐	☐	☐	☐	☐
**8. How often in the past 2 weeks have you found eating to be a pleasure?**	Never	A little of the time	Some of the time	Most of the time	All of the time
	☐	☐	☐	☐	☐
**9. Because of your illness, to what extent have you restricted the kinds of foods you eat?**	Very much	Much	Somewhat	A little	Not at all
	☐	☐	☐	☐	☐
**10. During the past 2 weeks, how well have you been able to cope with everyday streses?**	Extremely poorly	Poorly	Moderately	Well	Extremely well
	☐	☐	☐	☐	☐

Please continue on the next page

**11. How often during the past 2 weeks have you been sad about being ill?**	All of the time	Most of the time	Some of the time	A little of the time	Never
	☐	☐	☐	☐	☐
**12. How often during the past 2 weeks have you been nervous or anxious about your illness?**	All of the time	Most of the time	Some of the time	A little of the time	Never
	☐	☐	☐	☐	☐
**13. How often during the past 2 weeks have you been happy about life in general?**	Never	A little of the time	Some of the time	Most of the time	All of the time
	☐	☐	☐	☐	☐
**14. How often during the past 2 weeks have you been frustrated about your illness?**	All of the time	Most of the time	Some of the time	A little of the time	Never
	☐	☐	☐	☐	☐
**15. How often during the past 2 weeks have you been tired or fatigued?**	All of the time	Most of the time	Some of the time	A little of the time	Never
	☐	☐	☐	☐	☐
**16. How often during the past 2 weeks have you felt unwell?**	All of the time	Most of the time	Some of the time	A little of the time	Never
	☐	☐	☐	☐	☐
**17. Over the past week, have you woken up in the night?**	Every night	5-6 nights	3-4 nights	1-2 nights	Never
	☐	☐	☐	☐	☐
**18. Have you been troubled by changes in your appearance because of your illness?**	A great deal	A moderate amount	Somewhat	A little bit	Not at all
	☐	☐	☐	☐	☐
**19. How much physical strength do you feel you have lost because of your illness?**	A great deal	A moderate amount	Somewhat	A little bit	Not at all
	☐	☐	☐	☐	☐
**20. How much endurance have you lost because of your illness?**	A great deal	A moderate amount	Somewhat	A little bit	Not at all
	☐	☐	☐	☐	☐
**21. Because of your illness, to what extent do you feel unfit?**	Extremely unfit	Moderately unfit	Somewhat unfit	A little unfit	Fit
	☐	☐	☐	☐	☐

Please continue on the next page

**22. During the past 2 weeks, how often have you been able to complete your normal daily activities (school, work, household)?**	All of the time	Most of the time	Some of the time	A little of the time	Never
	☐	☐	☐	☐	☐
**23. During the past 2 weeks, how often have you been able to take part in your usual patterns of leisure or recreational activities?**	All of the time	Most of the time	Some of the time	A little of the time	Never
	☐	☐	☐	☐	☐
**24. During the past 2 weeks, how much have you been troubled by the medical treatment of your illness?**	Very much	Much	Somewhat	A little	Not at all
	☐	☐	☐	☐	☐
**25. To what extent have your personal relations with people close to you (family and friends) worsened because of your illness?**	Very much	Much	Somewhat	A little	Not at all
	☐	☐	☐	☐	☐
**26. To what extent has your sexual life been impaired because of your illness?**	Very much	Much	Somewhat	A little	Not at all
	☐	☐	☐	☐	☐
**27. How often in the past 2 weeks have you been troubled by liquid or food coming up into your mouth (regurgitation)?**	All of the time	Most of the time	Some of the time	A little of the time	Never
	☐	☐	☐	☐	☐
**28. How often during the past 2 weeks have you felt uncomfortable because of a slow speed of eating?**	All of the time	Most of the time	Some of the time	A little of the time	Never
	☐	☐	☐	☐	☐
**29. How of then during the past 2 weeks have you had trouble swallowing your food?**	All of the time	Most of the time	Some of the time	A little of the time	Never
	☐	☐	☐	☐	☐
**30. How often during the past 2 weeks have you been troubled by urgent bowel movements?**	All of the time	Most of the time	Some of the time	A little of the time	Never
	☐	☐	☐	☐	☐
**31. How often during the past 2 weeks have you been troubled by diarrhoea (loose bowel movements)?**	All of the time	Most of the time	Some of the time	A little of the time	Never
	☐	☐	☐	☐	☐

Please continue on the next page

**32. How often during the past 2 weeks have you been troubled by constipation (hard stools)?**	All of the time	Most of the time	Some of the time	A little of the time	Never
	☐	☐	☐	☐	☐
**33. How often during the past 2 weeks have you been troubled by nausea (feeling sick)?**	All of the time	Most of the time	Some of the time	A little of the time	Never
	☐	☐	☐	☐	☐
**34. How often during the past 2 weeks have you been troubled by blood in the stool?**	All of the time	Most of the time	Some of the time	A little of the time	Never
	☐	☐	☐	☐	☐
**35.. How often during the past 2 weeks have you been troubled by heartburn?**	All of the time	Most of the time	Some of the time	A little of the time	Never
	☐	☐	☐	☐	☐
**36. How often during the past 2 weeks have you been troubled by uncontrolled stools (incontinence)?**	All of the time	Most of the time	Some of the time	A little of the time	Never
	☐	☐	☐	☐	☐

Is there anything further you would wish to tell us about?

SF-36 Survey: This survey asks for your views about your health. This information will help keep track of how you feel and how well you are able to do your usual activities. For each of the following questions, please select the number that best describes your answer.

**1. In general, would you say your health is:**	Excellent	Very Good	Good	Fair	Poor
	☐ 1	☐ 2	☐ 3	☐ 4	☐ 5
**2. Compared to one year ago, your health is:**	Much better than one year ago	Somewhat better than one year ago	About the same	Somewhat worse than one year ago	Much worse now than one year ago
	☐ 1	☐ 2	☐ 3	☐ 4	☐ 5

**3. The following items are about activities you might do during a typical day. <u>Does your health now limit you in these activities? If so, how much?</u>**			
a. Vigorous activities, such as running, lifting heavy objects, participating in strenuous sports	Yes, limited a lot	Yes, limited a little	No, Not limited at all
	☐ 1	☐ 2	☐ 3
b. Moderate activities, such as moving a table, pushing a vacuum cleaner, bowling, or playing golf	Yes, limited a lot	Yes, limited a little	No, Not limited at all
	☐ 1	☐ 2	☐ 3
c. Lifting or carrying groceries	Yes, limited a lot	Yes, limited a little	No, Not limited at all
	☐ 1	☐ 2	☐ 3
d. Climbing several flights of stairs	Yes, limited a lot	Yes, limited a little	No, Not limited at all
	☐ 1	☐ 2	☐ 3
e. Climbing one flight of stairs	Yes, limited a lot	Yes, limited a little	No, Not limited at all
	☐ 1	☐ 2	☐ 3
f. Bending, kneeling, or stooping	Yes, limited a lot	Yes, limited a little	No, Not limited at all
	☐ 1	☐ 2	☐ 3
g. Walking more than a mile	Yes, limited a lot	Yes, limited a little	No, Not limited at all
	☐ 1	☐ 2	☐ 3
h. Walking several blocks	Yes, limited a lot	Yes, limited a little	No, Not limited at all
	☐ 1	☐ 2	☐ 3

Please continue on the next page

i. Walking one block	Yes, limited a lot	Yes, limited a little	No, Not limited at all
	☐ 1	☐ 2	☐ 3
j. Bathing or dressing yourself	Yes, limited a lot	Yes, limited a little	No, Not limited at all
	☐ 1	☐ 2	☐ 3

**4. During the past 4 weeks, have you had any of the following problems with your work or other regular daily activities as a result of your physical health?**		
a. Cut down the amount of time you spent on work or other activities	☐ Yes	☐ No
b. Accomplished less than you would like	☐ Yes	☐ No
c. Were limited in the kind of work or other activities	☐ Yes	☐ No
d. Had difficulty performing the work or other activities (for example, it took extra effort)	☐ Yes	☐ No
**5. During the past 4 weeks, have you had any of the following problems with your work or other regular daily activities as a result of any emotional problems (such as feeling depressed or anxious)?**		
a. Cut down the amount of time you spent on work or other activities	☐ Yes	☐ No
b. Accomplished less than you would like	☐ Yes	☐ No
c. Didn't do work or other activities as carefully as usual	☐ Yes	☐ No

**6. During the past 4 weeks, to what extent has your physical health or emotional problems interfered with your normal social activities with family, friends, neighbors, or groups?**	Not at all	Slightly	Moderately	Quite a bit	Extremely
	☐ 1	☐ 2	☐ 3	☐ 4	☐ 5

**7. How much bodily pain have you had during the past 4 weeks?**	None	Very mild	Mild	Moderate	Severe	Very severe
	☐ 1	☐ 2	☐ 3	☐ 4	☐ 5	☐ 6

Please continue on the next page

**8. During the past 4 weeks, how much did pain interfere with your normal work (including both work outside the home and housework)?**	Not at all	A little bit	Moderately	Quite a bit	Extremely
	☐ 1	☐ 2	☐ 3	☐ 4	☐ 5

These questions are about how you feel and how things have been with you during the past 4 weeks. For each question, please give the one answer that comes closest to the way you have been feeling. (Select One Number on Each Line)

9. How much of the time during the past 4 weeks . . .						
a. Did you feel full of energy?	All of the Time	Most of the Time	A Good Bit of the Time	Some of the Time	A Little of the Time	None of the Time
	☐ 1	☐ 2	☐ 3	☐ 4	☐ 5	☐ 6
b. Have you been a very nervous person?	All of the Time	Most of the Time	A Good Bit of the Time	Some of the Time	A Little of the Time	None of the Time
	☐ 1	☐ 2	☐ 3	☐ 4	☐ 5	☐ 6
c. Have you felt so down in the dumps that nothing could cheer you up?	All of the Time	Most of the Time	A Good Bit of the Time	Some of the Time	A Little of the Time	None of the Time
	☐ 1	☐ 2	☐ 3	☐ 4	☐ 5	☐ 6
d. Have you felt calm and peaceful?	All of the Time	Most of the Time	A Good Bit of the Time	Some of the Time	A Little of the Time	None of the Time
	☐ 1	☐ 2	☐ 3	☐ 4	☐ 5	☐ 6
e. Did you have a lot of energy?	All of the Time	Most of the Time	A Good Bit of the Time	Some of the Time	A Little of the Time	None of the Time
	☐ 1	☐ 2	☐ 3	☐ 4	☐ 5	☐ 6
f. Have you felt downhearted and blue?	All of the Time	Most of the Time	A Good Bit of the Time	Some of the Time	A Little of the Time	None of the Time
	☐ 1	☐ 2	☐ 3	☐ 4	☐ 5	☐ 6
g. Did you feel worn out?	All of the Time	Most of the Time	A Good Bit of the Time	Some of the Time	A Little of the Time	None of the Time
	☐ 1	☐ 2	☐ 3	☐ 4	☐ 5	☐ 6

Please continue on the next page

h. Have you been a happy person?	All of the Time	Most of the Time	A Good Bit of the Time	Some of the Time	A Little of the Time	None of the Time
	☐ 1	☐ 2	☐ 3	☐ 4	☐ 5	☐ 6
i. Did you feel tired?	All of the Time	Most of the Time	A Good Bit of the Time	Some of the Time	A Little of the Time	None of the Time
	☐ 1	☐ 2	☐ 3	☐ 4	☐ 5	☐ 6

**10. During the past 4 weeks, how much of the time has your physical health or emotional problems interfered with your social activities (like visiting with friends, relatives, etc.)?**	All of the time	Most of the time	Some of the time	A little of the time	None of the time
	☐ 1	☐ 2	☐ 3	☐ 4	☐ 5

**11. How TRUE or FALSE is each of the following statements for you.**					
a. I seem to get sick a little easier than other people	Definitely True	Mostly True	Don't Know	Mostly False	Definitely False
	☐ 1	☐ 2	☐ 3	☐ 4	☐ 5
b. I am as healthy as anybody I know	Definitely True	Mostly True	Don't Know	Mostly False	Definitely False
	☐ 1	☐ 2	☐ 3	☐ 4	☐ 5
c. I expect my health to get worse	Definitely True	Mostly True	Don't Know	Mostly False	Definitely False
	☐ 1	☐ 2	☐ 3	☐ 4	☐ 5
d. My health is excellent	Definitely True	Mostly True	Don't Know	Mostly False	Definitely False
	☐ 1	☐ 2	☐ 3	☐ 4	☐ 5

Is there anything further you would wish to tell us about?

Long-Term Outcomes in Hirschsprung's Disease (GOSH)
Questionnaire for adult patients
V1.1 18/09/2017

Male sexual function questions – Please remember your responses will be treated with the utmost confidentiality.

1. Are you currently in a stable relationship?

☐ Yes / ☐ No

2. Have you had sexual intercourse? (if not please continue to question 6.)

☐ Yes / ☐ No

If yes, first at age years

3. Have you tried to have children with your partner?

☐ No
☐ Yes, but I have not yet conceived (fallen pregnant)
☐ Yes, we have fallen pregnant times

4. Do you have biological children?

☐ Yes, children
☐ No

Additional information (e.g. if you have biological children with other than your current spouse/partner):

5. Have you needed any fertility investigations or treatment?

☐ No, we have not needed them (no problems conceiving)
☐ No, I have not tried to have a baby
☐ No, but we are considering going for investigations
☐ Yes

Please give details:

6. Are you able to climax (have an orgasm) from sexual stimulation?

☐ Never
☐ Rarely
☐ Sometimes
☐ Usually/Always

6. Do you ejaculate when you orgasm?

☐ Yes
☐ No – absent or dry ejaculations

7. EHS (Erectile Hardness Score

How would you rate your erections/erectile function (tick box that applies)

☐ Penis is completely hard and fully rigid
☐ Penis is hard enough for penetration, but not completely hard
☐ Penis is hard, but not hard enough for penetration.
☐ Penis is larger, but not hard
☐ Penis does not enlarge.

8. Male sexual function index (MSFI)

Please circle the most appropriate answer:

	completely agree	moderately agree	slightly agree	slightly disagree	moderately disagree	completely disagree
1. When I think about my sexual life, I feel frustrated	☐	☐	☐	☐	☐	☐
2. When I think about my sexual life, I feel depressed	☐	☐	☐	☐	☐	☐
3. When I think about my sexual life, I feel like less of a man	☐	☐	☐	☐	☐	☐
4. I have lost confidence in myself as a sexual partner	☐	☐	☐	☐	☐	☐
5. When I think about my sexual life, I feel anxious	☐	☐	☐	☐	☐	☐
6. When I think about my sexual life, I feel angry	☐	☐	☐	☐	☐	☐
7. I worry about the future of my sexual life	☐	☐	☐	☐	☐	☐
8. When I think about my sexual life, I am embarrassed	☐	☐	☐	☐	☐	☐
9. When I think about my sexual life, I feel guilty	☐	☐	☐	☐	☐	☐
10. When I think about my sexual life, I worry that my partner feels hurt or rejected	☐	☐	☐	☐	☐	☐
11. When I think about my sexual life, I feel like I have lost something	☐	☐	☐	☐	☐	☐

Is there anything further you would wish to tell us about?

That is the end of the questionnaire, thank you for taking part.
