# Supplementary figures and images for: Transitional Care in Patients With Hirschsprung Disease: Those Left Behind
Source: Dis Colon Rectum. 2024 Apr 23;67(7):977–84. doi: 10.1097/DCR.0000000000003208 (PMC11163890; doi:10.1097/DCR.0000000000003208)

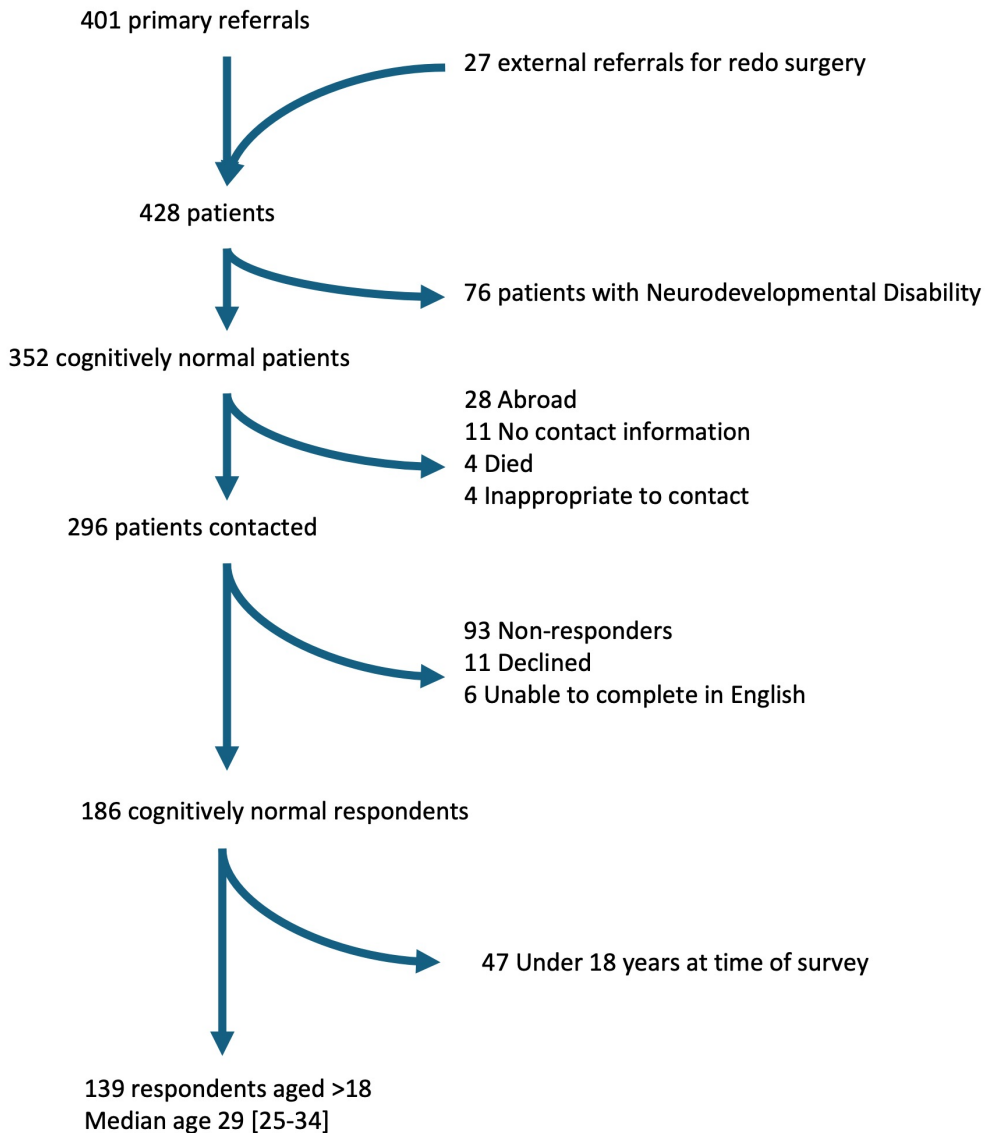

Supplement: Supplementary file 4 [file dcr-67-977-s004.pdf]
